# Thyrotoxicosis after COVID-19 Infection with a Delay in Graves' Disease Antibody Positivity

**DOI:** 10.1155/2023/8402725

**Published:** 2023-04-08

**Authors:** Dennis C. Boyle, Jamie A. Mullally

**Affiliations:** ^1^Westchester Medical Center, Department of Medicine, Section of Internal Medicine, Valhalla, USA; ^2^Westchester Medical Center, Department of Medicine, Division of Endocrinology and Metabolism, Valhalla, USA

## Abstract

*Background and Objective*. Mounting evidence implicates COVID-19 as a cause of thyroid dysfunction, including thyrotoxicosis due to both thyroiditis and Graves' disease (GD). In this report, we present a case of thyrotoxicosis following COVID-19 infection that was ultimately found to represent GD with significantly delayed diagnostic serum antibody positivity. *Case Report*. A 65-year-old woman with a history of uncomplicated COVID-19 infection one month prior, presented to the Emergency Department with exertional dyspnea and palpitations, and was found to be in atrial fibrillation with rapid ventricular response (AF with RVR). Labs showed subclinical hyperthyroidism and the patient was started on a beta-blocker and methimazole. One month later, thyroid-stimulating immunoglobulin (TSI) resulted negative and thyroid function tests had normalized. The clinical picture suggested thyroiditis, and methimazole was stopped. One month later, the patient again presented in AF with RVR, with labs showing overt biochemical thyrotoxicosis. Antibodies were re-tested, and the thyrotropin receptor antibody (TRAb) and TSI resulted positive, confirming GD. *Discussion*. Most notable in this case is the feature of delayed GD antibody positivity: the diagnostic immunoassay for GD resulted negative one and two months after infection, but was ultimately positive three months after infection. To the authors' knowledge, this represents the longest delayed antibody positivity reported to date, amongst cases of new-onset GD following COVID. *Conclusion*. The clinical course of GD following COVID-19 infection is highly variable. This case underscores the need for vigilance in monitoring for delayed GD antibody positivity due to the important therapeutic implications of distinguishing thyroiditis from GD.

## 1. Introduction

Mounting evidence implicates COVID-19 as a cause of thyroid dysfunction, including thyrotoxicosis due to both thyroiditis and Graves' disease (GD). GD is a form of autoimmune hyperthyroidism that is mediated by autoantibody stimulation of the thyrotropin receptor. It is known to occur in genetically-predisposed individuals who are exposed to environmental stressors – including viral or bacterial infection, disruption in iodine homeostasis, exposure to certain medications, radiation, and physiologic stress[1]. Numerous recent case reports have implicated COVID-19 as a cause of both new-onset – and relapse of dormant – Graves' disease [2–7].

We present a case of GD developing shortly after uncomplicated COVID-19 infection in a patient with no prior history of endocrinopathy or autoimmune disease, but with a notable delay in time to GD antibody-positivity.

## 2. Case Presentation

A 65-year-old female with a history of uncomplicated COVID-19 infection presented to the Emergency Department with progressive exertional dyspnea and palpitations for two weeks. The patient endorsed one episode of atypical chest pain; relevant review of systems was otherwise negative.

On physical exam, she was found to have vital signs of temperature 36.4°C, heart rate 138 beats per minute, blood pressure 148/118 mmHg, respiratory rate 22, SpO_2_ 97%. She was not in acute distress but was short of breath when speaking in full sentences. Her thyroid was nontender, not enlarged, and without palpable nodularity.

EKG showed AF with RVR (ventricular rate 142 bpm). Transthoracic echocardiography showed left atrial dilation and was otherwise normal. Initial labs revealed TSH 0.004 mIU/L (Nl 0.350-4.700 mIU/L). Thyroid ultrasound showed a homogenous gland without asymmetry, nodules or focal lesions, and normal vascularity on color flow Doppler. She underwent a CT scan with iodinated contrast to rule out PE, and so was unable to immediately undergo a thyroid uptake and scan. COVID-19 nasal swab PCR was negative.

The patient had no prior history of thyroid disease, other endocrinopathy, autoimmune disease, arrhythmia, coronary artery disease or cardiomyopathy. Her COVID-19 infection had occurred one month prior to presentation to the ED; at that time, she had symptoms of cough, exertional dyspnea, and sinus congestion – which resolved entirely.

With a very low TSH and signs of severe thyrotoxicosis with the potential for impending thyroid storm (Burch Wartofsky score = 35), the patient was started on metoprolol and empiric methimazole while awaiting additional diagnostic studies. Thereafter, she spontaneously converted to normal sinus rhythm (NSR). Additional labs showed total T4 (TT4) 8.7 mcg/dL (Nl 4.5-12.0 mcg/dL), free T4 1.3 ng/dL (Nl 0.7-1.9 ng/dL), and total T3 (TT3) 92.8 ng/dL (Nl 79.0-149.0 ng/dL). Thyroid-stimulating immunoglobulin (TSI) was pending at the time of discharge and she was continued on daily methimazole 15 mg and metoprolol succinate 200 mg.

She followed up with endocrinology and cardiology three weeks after discharge at which time TSH had normalized, and TT4 and TT3 were slightly low. TSI from the initial hospitalization had come back negative (Table 1). EKG showed NSR at 69 beats per minute. The etiology of the low TSH was thought to be thyroiditis, likely related to COVID-19 infection, and methimazole was discontinued. A thyrotropin receptor antibody (TRAb) was also sent at this time and resulted normal at 1.20 IU/L (Nl 0.00–1.75).

She followed up again one month later, at which time she complained of exertional dyspnea and palpitations and was again found to be in AF with RVR. She was sent to the Emergency Department and was admitted to Cardiology. At that point, labs showed an undetectable TSH with high total T4, free T4, and total T3 (TSH < 0.002 mIU/L, total T4 13.7 mcg/dL [Nl 4.5-12.0], free T4 2.1 ng/dL [Nl 0.7-1.9], and total T3 167.0 ng/dL [Nl 79-149]). At this time, she was found to have an elevated TRAb of 2.50 IU/L (Nl 0.00-1.75) and TSI of 0.58 IU/L (Nl 0.00-0.55). Methimazole, at doses of 15-20 mg daily, was resumed for treatment of thyrotoxicosis. The AF with RVR was difficult to control and required increasing doses of several rate and rhythm control medications (metoprolol, digoxin, and dofetilide), as well as numerous attempted electrical cardioversions, which ultimately achieved return to NSR.

## 3. Discussion

In this report, we have presented a case of new-onset Graves' disease occurring in close temporal relationship with mild COVID-19 infection. Our case is noteworthy because the patient initially presented with subclinical hyperthyroidism with negative TSI and TRAb and an unremarkable thyroid ultrasound with normal color flow Doppler, and only later developed overt thyrotoxicosis and positive TSI and TRAb. It is important to recognize there can be a delay in antibody positivity in patients with overt thyrotoxicosis after COVID-19 infection, and to follow patients closely so as not to confuse a diagnosis of thyroiditis and GD, as the management is inherently different. Thyroiditis is a self-limited process involving the release of pre-formed thyroid hormone that can be treated with anti-inflammatory medications but does not require anti-thyroidal medication [8]. In contrast, GD involves autoantibody stimulation of the thyrotropin receptor, resulting in excess thyroid hormone synthesis. GD requires treatment with anti-thyroid medication, radioactive iodine, or surgery. While this patient may have developed post-COVID-19 Graves' disease with autoantibody levels that were not initially at a detectable level, a possible alternative explanation is that the patient initially had thyroiditis and only later developed Graves' disease.

Viral infection has long been known to play an important role in the pathogenesis of autoimmune thyroid disease, including both GD and Hashimoto's thyroiditis [1, 9]. While a direct causal link between COVID-19 infection and GD has not been established, a temporal relationship has been noted in numerous case reports—including both new onset [2–5, 10–12] and relapse of previously stable [2, 6, 12, 13] Graves' disease. Tutal et al., in a systematic review published in 2022, presented 14 cases of GD that occurred shortly after COVID-19 infection; of those cases, 6 involved new-onset GD, while 8 occurred in patients in stable remission [7]. In that review, cases of autoimmune thyroiditis presented concurrently with COVID-19 infection or within two months of the infection. Inaba and Aizawa found that new-onset GD tended to present 6–8 weeks following COVID-19 infection [14]. Our patient initially presented with subclinical thyrotoxicosis and negative antibodies for GD four and seven weeks post-COVID infection. Her antibodies were positive approximately 12 weeks post-COVID infection, highlighting the need to be vigilant for a delayed GD diagnosis even with initially negative antibodies.

An elevated TRAb or TSI confirms the diagnosis of GD. A 2012 meta-analysis of 111 publications on diagnostic assays found 3rd generation TRAb immunoassays to have pooled sensitivity and specificity of 98.3% and 99.2%, respectively (vs. 2nd gen. with 97.1% and 97.4%) [15]. TRAb can be considered on the basis of their effect on the thyrotropin receptor: i.e., stimulating, blocking, and apoptosis-inducing. The presence of the former, also known as thyroid-stimulating immunoglobin (TSI), can also be used to confirm GD diagnosis. A 2021 analysis of 324 subjects with GD compared a 3rd generation TRAb immunoassay with a TSI immunoassay on the basis of receiver operating characteristic curves and found the two tests to have comparable efficacy [16].

It is widely agreed that GD pathogenesis is multifactorial—involving both a genetic predisposition and environmental influences [1, 17]. It has traditionally been considered that an imbalance between T helper (Th) 1 and Th2 cells drives the production of autoantibodies (e.g., thyroid-stimulating immunoglobulin) by driving Th2-mediated humoral immunity [1, 16]. More recent studies suggest the coinvolvement of other T helper cell subsets, including Th17, Th22, and T follicular helper cells [17]. Specific cytokines also likely play a role; for example, IL-6 system activation and increased serum IL-6 receptor concentrations have long been known to occur in patients with active GD [18]. Interestingly, the elevation of this proinflammatory cytokine is also associated with severe COVID-19. Moreover, blockade of the IL-6 receptor (via tocilizumab and sarilumab) and direct IL-6 inhibition (via siltuximab) have played an important role in the treatment of patients with severe COVID-19 [19].

A 2021 review by Murugan et al. discusses the pathogenesis of thyroid diseases related to COVID-19 infection [20]. The authors posit that the virus directly infects thyroid cells and adjunctively induces its effects through cytokines and other mediators. In the case of direct infection, the ACE2 protein, which is prominently expressed in both pulmonary and thyroid tissue, is used by the virus to gain cellular entry. Interestingly, the ACE2 protein is shown to be overexpressed in patients with hyperthyroidism. The authors propose a multifactorial pathogenesis of COVID-19-induced GD. In addition to direct cellular infection, a “COVID-19-induced cytokine storm” produces an inflammatory state that further predisposes to the future development of autoimmune thyroid disease, such as GD.

## 4. Conclusion

In the present article, we have documented a case of new-onset Graves' disease following COVID-19. Most notable in this case is the feature of delayed GD antibody positivity: the diagnostic immunoassay for GD resulted negative one and two months after infection, but was ultimately positive three months after infection. To the authors' knowledge, this represents the longest delay in antibody positivity reported to date, amongst cases of new-onset GD following COVID-19. Our findings serve to support the growing body of evidence suggesting an association between COVID-19 and GD. Further, this case of thyrotoxicosis following COVID-19 infection underscores the need for clinicians to be aware of GD presenting with delayed antibody positivity – given important differences in management in comparison to thyroiditis.

## Figures and Tables

**Table 1:** Laboratory thyroid function tests.

	Reference range	# Weeks from COVID-19			
		4	7	11	12
TSH (mIU/L)	0.35–4.70	**0.004**	1.180	**<0.002**	**<0.002**
Free T4 (ng/dL)	0.7–1.9	1.2	0.8	**2.1**	1.3
Total T4 (mcg/dL)	4.5–12.0	8.7	**4.2**	**13.7**	
Free T3 (pg/mL)	2.8–4.4	2.8		**6.5**	3.9
Total T3 (ng/dL)	79–149	92.8	**68.4**	**167.0**	120
TRAb (IU/L)	0–1.75		1.20		**2.50**
TSI (IU/L)	0–0.55	0.16		**0.58**	
TPO Ab (IU/mL)	0.0–9.0			<10	**18**

TSH = thyroid-stimulating hormone; TRAB = thyrotropin receptor antibody; TSI = thyroid-stimulating immunoglobulin; TPO = thyroid peroxidase. Abnormal values are in bold.

## Data Availability

The data used to support the findings of this study are included within the article.

## Abbreviations

GD: Graves' disease
TSH: Thyroid-stimulating hormone
TSI: Thyroid-stimulating immunoglobulin
TRAb: Thyrotropin receptor antibody
TPO: Thyroid peroxidase
T4: Thyroxine
T3: Triiodothyronine
TT4: Total T4
TT3: Total T3
AF with RVR: Atrial fibrillation with rapid ventricular response
NSR: Normal sinus rhythm
Th: T helper
IL: Interleukin.
